# The WT1/MVP-Mediated Stabilization on mTOR/AKT Axis Enhances the Effects of Cisplatin in Non-small Cell Lung Cancer by a Reformulated Yu Ping Feng San Herbal Preparation

**DOI:** 10.3389/fphar.2018.00853

**Published:** 2018-08-07

**Authors:** Jian-Shu Lou, Yi-Teng Xia, Huai-You Wang, Xiang-Peng Kong, Ping Yao, Tina T. X. Dong, Zhong-Yu Zhou, Karl W. K. Tsim

**Affiliations:** ^1^College of Pharmaceutical Science, Zhejiang Chinese Medical University, Hangzhou, China; ^2^Shenzhen Key Laboratory of Edible and Medicinal Bioresources, SRI, The Hong Kong University of Science and Technology, Shenzhen, China; ^3^Division of Life Science, Center for Chinese Medicine, The Hong Kong University of Science and Technology, Kowloon, Hong Kong; ^4^Key Laboratory of Plant Resources Conservation and Sustainable Utilization, Guangdong Provincial Key Laboratory of Applied Botany, South China Botanical Garden, Chinese Academy of Sciences, Guangzhou, China

**Keywords:** non-small cell lung cancer, MVP, mTORC2, WT1, herbal medicine, chemo-resistance

## Abstract

Chemo-resistance is an obstacle in therapy of lung cancer. Alternative therapy of using herbal medicine has been proposed to resolve this obstacle. Yu Ping Feng San (YPFS), a common Chinese herbal medicinal mixture, has been reported to show anti-drug resistance on cisplatin (DDP), a common lung cancer drug. To optimize the anti-cancer function of YPFS, different Chinese herbal extracts having known function to overcome lung cancer were screened in combining with YPFS, as to increase the efficacy of DDP in drug resistance lung cancer cell, A549/DDP. Amongst these herbal extracts, Ginkgo Folium exhibited the most promoting sensitized effect. This revised herbal formula, named as YPFS_+GF_, promoted the DDP-induced toxicity by over 2-fold as compared to that of YPFS alone; this potentiation was confirmed by inducing cell apoptosis. The anti-drug resistance of YPFS, triggered by an increase of intracellular concentration of DDP, was accompanied by an increased expression and activity of WT1, which consequently decreased the transcript level of MVP. In addition, the MVP-mediated downstream effector mTOR2/AKT was disrupted after application of YPFS_+GF_ in DDP-treated A549/DDP cell: this disruption was characterized by the decline of mTORC2 components, e.g., Rictor, p-mTOR, as well as the phosphorylation level of its downstream protein AKT. The disruption on mTORC2/AKT could be reversed by mTORC2 inducer insulin and promoted by mTORC2 inhibitor PP242. Thus, the anti-drug resistance of YPFS_+GF_ in DDP-treated lung cancer cells might be mediated by the down regulation of WT1/MVP axis, as well as the downstream anti-apoptotic pathway of mTORC2/AKT signaling. Herbal medicine is one of the main adjuvant therapies in non-small cell lung cancer, and this novel herbal formula supports the prescription of traditional Chinese medicine in cancer treatment.

## Introduction

Non-small cell lung cancer is the major type of lung cancer accounting approximately 80% of lung cancer patients today (Haghgoo et al., 2015). Nowadays, either classic chemotherapeutic drug or target therapeutic drug inevitably exhibits resistance after a period of drug treatment. DDP is one of the first line treatments of chemotherapeutic drugs for NSCLC in clinic for decades. The key flaw of DDP is late required resistance, which enables cancer cells to evade apoptotic death, consequently leading to poor response in cancer patients (Waissbluth and Daniel, 2013).

Multiple mechanisms are proposed to be involved in DDP resistance in cancer cell, including promoting cell growth and development, repairing DNA damage and stimulating endocytosis. The predominant feature responsible for DDP resistance is the declined intracellular drug accumulation, i.e., increase of drug efflux. Dozens of genes in cancer cell could contribute to intracellular distribution of DDP, e.g., multidrug resistant protein 1–3, breast cancer resistant protein, and MVP (Shen et al., 2012). MVP is a basic component of the vault complex and forms the outer shell of vault: this is over expressed in multidrug-resistant lung cancer cells. In line to this observation, MVP is being considered to be a biomarker of drug resistance (Berger et al., 2009). Because the hollow-barrel structure of MVP could able to open or close, and thus MVP has intracellular transport function (Steiner et al., 2006). MVP is also involving in several intracellular signal cascades, e.g., mTOR/AKT, EGFR/PI3K, as a result to promote drug resistance in cancer cells (Chung et al., 2005; Wojtowicz et al., 2017). Combination of DDP with other drugs is considered to be a good strategy to address drug resistance in cancer therapy; however, synthesized compounds usually exhibit stronger side effects. Under this scenario, the focus has been shifted to safe herbal products. Statistical analysis from clinical reports of Chinese medicine hospital during 1989 to 2007 indicated that the most frequently used medicinal herbs in lung carcinoma are those having function of replenishing “Qi” and nourishing “Yin” to lung.

Our previous study demonstrated that YPFS, a herbal mixture composes of Astragali Radix (Huangqi; the root of *Astragalus membranaceus* (Fisch.) Bunge or *Astragalus membranaceus* (Fisch.) Bunge var. *mongholicus* (Bunge) P.K. Hsiao), Atractylodis Macrocephalae Rhizoma (Baizhu; the rhizomes of *Atractylodes macrocephala* Koidz.) and Saposhnikoviae Radix [Fangfeng; the roots of *Saposhnikovia divaricata* (Turcz.) Schischk.], showed the reverse effect on DDP-induced resistance in NSCLC cell line A549, which was proposed to be acting through down regulation of MVP (Lou et al., 2016). Having the identification of YPFS in anti-cancer, we aimed to re-formulate the herbal mixture as to increase its efficiency in treating lung cancer.

According to traditional Chinese medicine (TCM) theory, lung adenocarcinoma is due to the deficiency of “*Lung Yin.*” Thus, the herbs having function in nourishing “*Yin*,” as described in TCM, should be our first choice to be included in YPFS. Fourteen Chinese herbs, including Ophiopogonis Radix, Adenophorae Radix, Rehmanniae Radix, Lilii Bulbus and Gonkgo Folium, were screened in combining with YPFS: the aim was to identify new herbal mixture having superior anti-drug resistance in cultured DDP resistant A549 cell (Wang and Jing, 2010). Inclusion of Ginkgo Folium extract in YPFS was revealed to have the highest potentiation effect, and therefore a new formulation of YPFS + Ginkgo Folium, named as YPFS_+GF_, was proposed here for further development.

## Materials and Methods

### Reagents, Cell Lines and Antibodies

The FITC-labeled Annexin V Apoptosis Detection Kit was obtained from BD Biosciences (San Jose, CA, United States). JC-1, DDP, PP242, and insulin were purchased from Sigma-Aldrich (St. Louis, MO, United States). The culture medium was obtained from Invitrogen Technologies (Carlsbad, CA, United States). The antibodies were obtained from the following sources: WT1 from Millipore (Billerica, MA, United States); MVP from Abcam (Cambridge, United Kingdom); mTOR, Rictor, p-AKT, cleaved-PARP, cleaved-caspase 3, horseradish peroxidase (HRP)-conjugated goat anti-rabbit antibody, HRP-conjugated goat anti-mouse antibody, and Alexa Fluor 555-conjugated goat anti-rabbit antibody from Cell Signaling Technology (Danvers, MA, United States). The water extracts of Rehmanniae Radix, Ophiopogonis Radix, Lilii Bulbus, Adenophorae Radix, Houttuyniae Herba, Polygonati Odorati Rhizoma, Dendrobii Caulis, Farfarae Flos, Platycodonis Radix, Fritillariae Cirrhosae Bulbus, Ginkgo Folium, Selaginellae Herba, Taxus Folium, Ilex Folium were obtained from Nanjing Ze Lang Biological Technology Co., Ltd. (Nanjing, China). Water extraction was being used here because of its common form of preparation, historically. A549 was purchased from American Type Culture Collection (ATCC, Manassas, VA, United States). A549/DDP (DDP resistance cell line) was obtained from the China Academy of Military Medical Science (Beijing, China). Cells were cultured in RPMI 1640 with 10% fetal bovine serum, 1% penicillin and 1% streptomycin. The cells were maintained in a humidified 5% CO_2_ atmosphere at 37°C.

### Herbal Preparation

In the preparation of YPFS, the herbal mixture of roots of *A. membranaceus* var. *mongholicus*, the rhizomes of *A. macrocephala* and the roots of *S. divaricata* in a weight ratio of 1:2:1 was boiled in 8 volumes of water (v/w) by heating for 2 h. The residues were then re-boiled in 6 volumes of water for 1 h. The two extracts were combined, filtered, dried by lyophilization and stored at 4°C (Du et al., 2014; Lou et al., 2016).

### Cytotoxicity and Apoptosis Detection

In cell viability assay, A549/DDP cells were seeded in 96-well plates at 3,000 cells per well. The cells were treated with DDP, DDP + YPFS, DDP + GF and DDP + YPFS + GF for 48 h. After incubation with MTT, medium was removed and dissolved in DMSO. The spectrophotometric absorbance at 570 nm was determined. For apoptosis assay, cultured A549/DDP cells were seeded and treated with DDP, YPFS, GF, YPFS_+GF_, DDP + GF, DDP + YPFS, and DDP + YPFS_+GF_. The apoptosis assay was conducted as previous described (Lou et al., 2016, 2017). Briefly, both floating and adherent cells were collected and washed with PBS. Cells were stained with annexin-V/FITC and propidium iodide for 15 min at room temperature in dark. The fluorescence was detected by flow cytometry with the acquisition criteria of 10,000 events for each sample, and the quadrants were set according to the population of viable, untreated samples. The data were analyzed using FACSAria equipped with the CellQuest Software (BD Biosciences).

### Detection of Intracellular DDP

The intracellular concentration of DDP was measured according to the previous study (Lou et al., 2016). Briefly, cells were treated with DDP, DDP + GF, DDP + YPFS, and DDP + YPFS_+GF_ for 6 h. The cells were washed, harvested, mineralized in 500 μL 70% HNO_3_ at 80°C overnight. After digesting, the solution was diluted with water. Platinum determination was performed using ICP-OES.

### RNA Isolation and Real-Time PCR

Total RNAs were extracted using RNAzol RT reagent and were reversed transcribed into cDNAs, as previously described (Chen et al., 2016). Briefly, the cells were collected and lysed with RNAzol RT reagent. Water was added to lysate, vortexed and centrifuged, the aqueous layer was collected. The RNA was precipitated and washed by ethanol, dried, re-suspended in RNAase free water, and then quantified by spectrometry. RNA was reverse transcribed by MMLV according to the manufacturer’s instructions. The following primers were used: 5′-GTC TTC GGG CCT GAG CTG GTG TCG-3′ (S) and 5′-CTT GGC CGT CTC TTG GGG GTC CTT-3′ (AS) for MVP; and 5′-AAC GGA TTT GGC CGT ATT GG-3′ (S) and 5′-CTT CCC GTT CAG CTC TGG G-3′ (AS) for GAPDH. Real-time PCR was performed using SYBR Green Master mix (Roche) by LightCycler^®^ Real-Time PCR system (Roche, Basel, Switzerland). The data were normalized to the amount of the GAPDH housekeeping genes.

### Western Blot Analysis

Western blot was performed as previously described (Lou et al., 2016). In brief, the cells were lysed, and their protein concentrations were measured using Bradford method. Equal amounts of proteins were separated by SDS-PAGE and then transferred to nitrocellulose membranes. The membranes were blocked and then probed with indicated primary antibodies. The blots were rinsed and then incubated with secondary antibodies. The reactive bands were visualized using ECL (Thermo Scientific) and then calibrated by Chemidoc Imaging System (Bio-Rad; Hercules, CA, United States).

### Immunofluorescence

Cultured A549/DDP cells were placed on glass coverslips and treated with DDP, YPFS, GF, YPFS_+GF_, DDP + GF, DDP + YPFS, and DDP + YPFS_+GF_ for 48 h. The cells were washed and fixed with 4% formaldehyde. The cells were blocked (1X PBS/5% BSA/0.3% Triton^TM^ X-100) for 1 h. Then, primary antibody was incubated overnight at 4°C, cells were washed, and incubated with Alexa Fluor 555-conjugated goat anti-rabbit secondary antibody for 2 h followed by DAPI nuclear staining (5 mg/mL) for 15 min. After washing, the images were taken using a Zeiss Laser Scanning Confocal Microscope (LSM7 DUO). To analyze the nuclear translocation of WT1, the co-localization coefficients were calculated using the Zeiss co-localization coefficient function software.

### Chromatin Immunoprecipitation

Formaldehyde cross-linking and immunoprecipitations were carried out by using ChIP kit (Abcam, Cambridge, MA, United States). Briefly, the cross-linkage of proteins to DNA was carried out by formaldehyde at room temperature for 15 min, then glycine was included to a final concentration of 125 mM. After washing with ice-cold PBS, the cells were resuspended in ChIP lysis buffer, and the lysate was sonicated to shear DNA to an average fragment size of 200–1,000 bp. Diluted chromatin solution was incubated with either anti-WT1 antibody, IgG or H3 antibody, overnight at 4°C with rotation. The DNA purification was carried out according to the manufacturer’s instructions. Then, the DNA was amplified for 35 cycles of PCR with the following primers: 5′-ACT TGG TCC CAT TTG TGT GAC-3′ (S) and 5′-GTC CCC AGT TCT CTC CCA TC-3′ (AS).

### Statistical Analysis and Other Assays

Statistical tests have been done by using one-way analysis of variance. Data were expressed as Mean ± SEM. Statistically significant changes were classified as significant (^∗^) where *p* < 0.05 and more significant (^∗∗^) where *p* < 0.01 as compared with control group.

## Results

### Ginkgo Folium Enhances the Effect of YPFS in DDP-Induced Cytotoxicity

**FIGURE 1:**
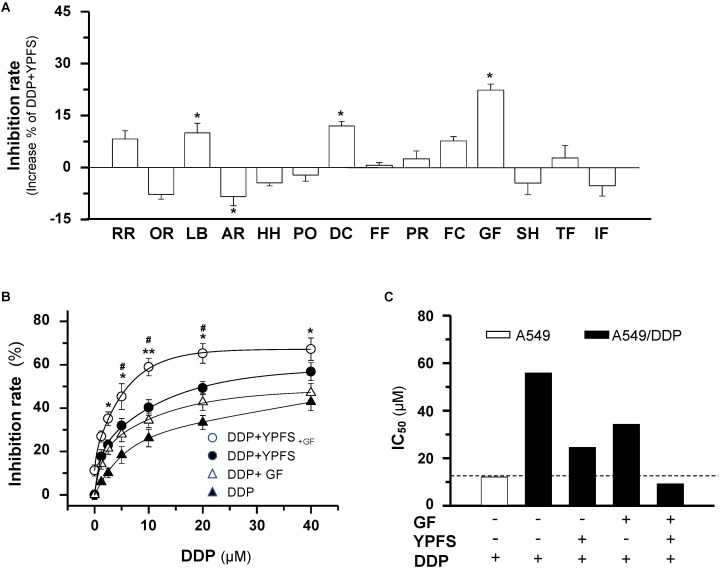
YPFS_+GF_ promotes YPFS-mediated sensitized effect in DDP-induced cytotoxicity on A549/DDP cells. **(A)**: Cells were seeded in 96-well plates (3 × 10^3^ cells/well) and allowed to adhere overnight and co-treated with DDP (10 μM ) and YPFS (1 mg/mL) or other re-formulae (1 mg/mL YPFS + 0.25 mg/mL herbal water extract) for 48 h, i.e., Rehmanniae Radix (RR), Ophiopogonis Radix (OR), Lilii Bulbus (LB), Adenophorae Radix (AR), Houttuyniae Herba (HH), Polygonati Odorati Rhizoma (PO), Dendrobii Caulis (DC), Farfarae Flos (FF), Platycodonis Radix (PR), Fritillariae Cirrhosae Bulbus (FC), Ginkgo Folium (GF), Selaginellae Herba (SH), Taxus Folium (TF), Ilex Folium (IF). Values are in percentage of increase on inhibition rate compared with that of DDP + YPFS combination. **(B)**: Cells were seeded as in **(A)**, DDP was applied at various concentrations in the absence or presence of YPFS (1 mg/mL), Ginkgo Folium (GF; 0.25 mg/mL) or YPFS_+GF_ (1 mg/mL + 0.25 mg/mL) for 48 h. Values are in the percentage of inhibition. **(C)**: The IC_50_ values of DDP on A549 and A549/DDP cells, as well as in the presence of YPFS, GF or YPFS_+GF_ in A549/DDP cells were shown. Each point represents the mean ± SEM, *n* = 3. ^∗^*p* < 0.05, ^∗∗^*p* < 0.01 versus DDP-treated alone, ^#^*p* < 0.05 versus the combined treatment of DDP and YPFS.

Firstly, the quality control of YPFS extract was demonstrated in a fingerprint (**Supplementary Figure 1A**). In addition, fifteen chemical markers within YPFS were determined by a rapid resolution liquid chromatography/tandem mass spectrometry, as previously reported (Du et al., 2014). The contents of these markers met the minimum requirements that established previously (**Supplementary Figure 1B**). The chemical standardization was to ensure the repeatability of all biochemical experiments. To increase the potency of YPFS, fourteen herbal water extracts, i.e., Rehmanniae Radix, Ophiopogonis Radix, Lilii Bulbus, Adenophorae Radix, Houttuyniae Herba, Polygonati Odorati Rhizoma, Dendrobii Caulis, Farfarae Flos, Platycodonis Radix, Fritillariae Cirrhosae Bulbus, Ginkgo Folium, Selaginellae Herba, Taxus Folium, Ilex Folium, were chosen to combine with YPFS in treating lung cancer cell. These chosen herbs are commonly used as Chinese medicine in cancer patients. All herbal extracts have no significantly cell inhibition effect at 0.25 mg/mL in cultured A549/DDP cells for 48 h of treatment (**Supplementary Figure 2**). One mg/mL of YPFS, a dose previously shown to achieve sub-maximal efficiency (Lou et al., 2016), was combined with the herbal extract at 0.25 mg/mL, a minimal dose routinely used in cell culture, and the mixed herbal extract was applied onto the DDP-treated cultured A549/DDP cells. Most of these herbal extracts showed no obvious promotion of YPFS in DDP-mediated anti-cancer effect. However, the extracts from Lilii Bulbus, Dendrobii Caulis and Ginkgo Folium showed the potentiation effect, and the best result was shown in case of Gingkgo Folium, having an increase of over 20% inhibition, as compared to that of YPFS + DDP (**Figure 1A**). The safety and potential benefits of Gingkgo Folium have been reported (Lou et al., 2017; Yuan et al., 2017). The novel herbal formula composed of YPFS + Gingkgo Folium extract in a weight ratio of 4:1, named as YPFS_+GF_, showed relatively high IC_50_ (29.2 mg/ml) in human umbilical vein endothelial (HUVEC) cells, and slightly decrease the cytotoxicity of DDP in HUVEC cells, suggesting the non-cytotoxicity of this herbal extract in culture (**Supplementary Figure 3**). Thus, YPFS_+GF_ was selected for further analyses. Application of YPFS_+GF_ in cultured A549/DDP cells notably strengthened the DDP-inhibited cell proliferation: A robust significant change was observed at 10 μM of DDP (**Figure 1B**). The resistance to DDP in A549/DDP cells was being characterized by the elevation of IC_50_, which was ∼4.6 folds higher than that of parental A549 cells (**Figure 1C**). The application of YPFS, or Ginkgo Folium extract, decreased the IC_50_ value; while the application of YPFS_+GF_ decreased the IC_50_ value much further by over 5-fold (**Figures 1B,C**). In probing the synergy of DDP and YPFS_+GF_ in A549/DDP cells, their combination index was calibrated under various conditions. Here, 10 μM DDP combined with YPFS_+GF_ exhibited the lowest combination index, i.e., the best synergy (**Supplementary Figure 4**). These data indicated that Gingkgo Folium could increase the sensitized effect of YPFS in DDP-inhibited cell proliferation.

**FIGURE 2:**
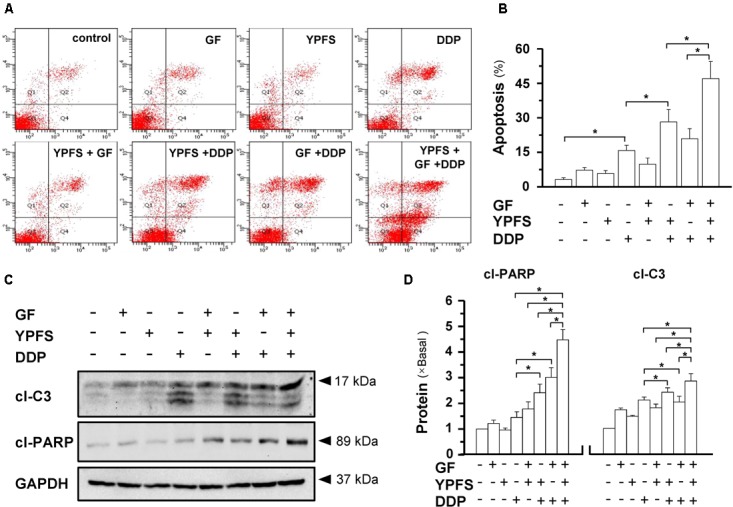
YPFS_+GF_ promotes the DDP-induced apoptosis in A549/DDP cells. **(A)**: Cultured A549/DDP cells were treated with YPFS (1 mg/mL), GF (0.25 mg/mL) or YPFS_+GF_ (1 mg/mL + 0.25 mg/mL) and DDP (10 μM) in absence or presence of YPFS (1 mg/mL), GF (0.25 mg/mL) or YPFS_+GF_ (1 mg/mL + 0.25 mg/mL) for 48 h. Cell apoptosis was detected by flow cytometry with Annexin V/PI apoptosis detection kit. The dual parametric dot plots combining annexin V-FITC and PI fluorescence showed the viable cell population in the bottom left quadrant (Q3), the early apoptotic cells in the bottom right quadrant (Q4), and the late apoptotic cells in the top right quadrant (Q2). **(B)**: Determination of apoptotic rates, as calibrated from **(A)**. Values are in percentage of apoptotic cell number. **(C)**: Western blot analyses of cleaved (cl)-caspase 3 at ∼17 kDa, and cleaved (cl)-PARP at ∼89 kDa. Expression of GAPDH (∼37 kDa) served as a control. **(D)**: Quantitation of cl-caspase 3, and cl-PARP, as calibrated from **(C)**. Values are in fold of change (X Basal) to control (no drug treatment). Each point represents the mean ± SEM, *n* = 3. ^∗^*p* < 0.05.

**FIGURE 3:**
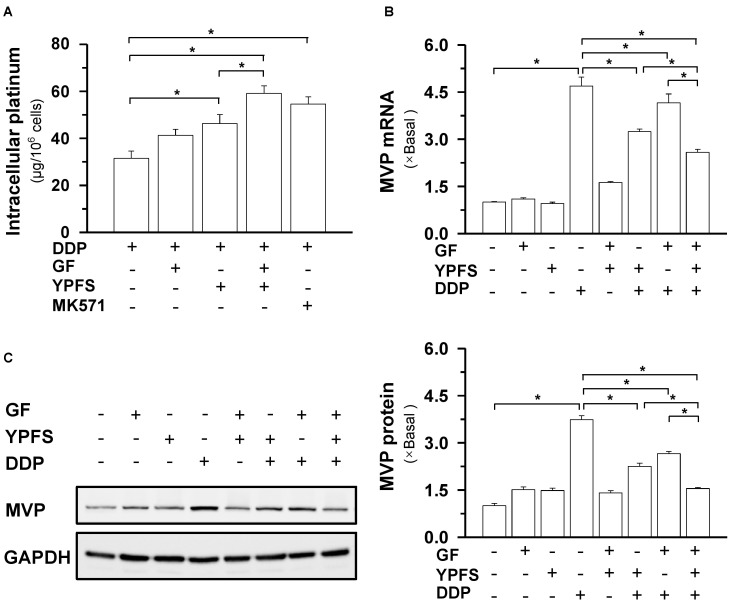
YPFS_+GF_ increases the intracellular concentration of DDP and decreases MVP expression in A549/DDP cells. **(A)**: Intracellular platinum (as of DDP in μg/ one million cells) was measured with ICP-OES. DDP at 100 μM, in absence or presence of YPFS (1 mg/mL), GF (0.25 mg/mL) or YPFS_+GF_ (1 mg/mL + 0.25 mg/mL), was applied onto A549/DDP cells for 6 h, then the cell lysate was collected for platinum determination. MK571 (50 μM), a MRP1 inhibitor, served as a control. **(B)**: Cultured A549/DDP cells were treated with DDP, YPFS, GF, YPFS_+GF_, as well as DDP with YPFS, GF or YPFS_+GF_ for 48 h, as in **Figure 2**. The amount of mRNA encoding MVP was determined by real-time PCR. Values are in fold of change (X Basal) to control (no drug treatment). **(C)**: The cell treatment was done as in **(B)**. The protein level of MVP (∼110 kDa) was determined by western blotting (left panel). Expression of GAPDH (∼37 kDa) served as a control. Quantitation of protein expression is shown in right panel. ^∗^*p* < 0.05, *n* = 3.

**FIGURE 4:**
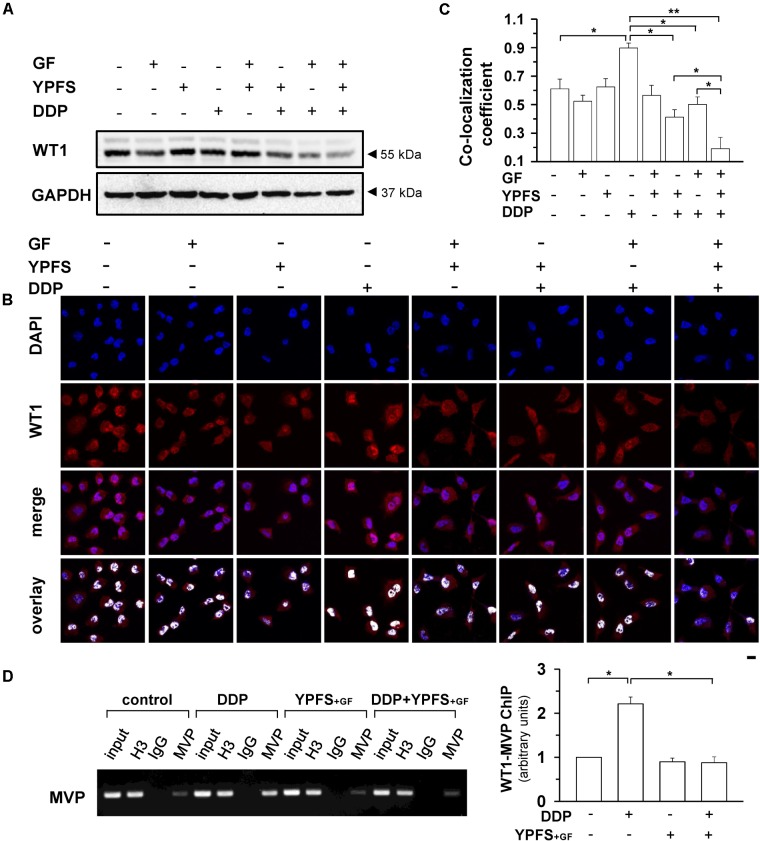
YPFS_+GF_ suppresses DDP-induced activation on WT1 in A549/DDP cells. **(A)**: Cultured A549/DDP cells were treated with DDP, YPFS, GF, YPFS_+GF_, as well as DDP with YPFS, GF or YPFS_+GF_ for 48 h, as in **Figure 2**. The protein level of WT1 (∼55 kDa) was determined by western blotting. Expression of GAPDH (∼37 kDa) served as a control. **(B)**: The cell treatment was done as in **(A)**. Immunofluorescence staining was applied to observe the localization of WT1 (red fluorescence) in nuclei (blue fluorescence) of A549/DDP cells. Bar = 20 μm. **(C)**: Co-localization coefficients were calculated by the co-localizing pixel for WT1 relative to the total number of pixels for the nuclei using Zeiss co-localization coefficient function software. **(D)**: Cultured A549/DDP cells were treated with DDP, YPFS_+GF_, as well as DDP with YPFS_+GF_ for 48 h. The binding of WT1 to MVP promotor (WT1-MVP) was detected by Chromatin immunoprecipitation (ChIP)/PCR assay (left panel). Quantitation is shown in right panel. Values are in fold of change (X Basal) to control (no drug treatment). Results are expressed as the mean ± SEM, *n* = 3. ^∗^*p* < 0.05, ^∗∗^*p* < 0.01.

### YPFS_+GF_ Increases DDP-Induced Apoptosis in A549/DDP Cell

The induction of cell apoptosis was analyzed in A549/DDP cells treated with DDP at 10 μM in presence or absence of herbal extracts. DDP by itself slightly increased the apoptosis rate in A549/DDP cells, and addition of YPFS further increased apoptosis rate (**Figures 2A,B**). Application of YPFS_+GF_ further elevated apoptosis rate by over 50%, double than that of only YPFS application. In western blot analysis, the apoptotic markers, cleaved caspase 3 and cleaved PARP, were markedly increased in the co-application of DPP + YPFS_+GF_: this apoptotic effect was significant higher than that of DPP treatment or DPP + YPFS treatment (**Figures 2C,D**). In contrast, the extract from Gingkgo Folium did not show such changes of apoptotic markers.

### YPFS_+GF_ Increases Intracellular DDP Through WT1/MVP Modulation

The key mechanism responsible for DDP-induced drug resistance is intracellular distribution of drug. Our previous study demonstrated that YPFS could notably increase the intracellular concentration of DDP in cultured A549/DDP cells. In drug resistance, the most functional target might be MVP, contributing to an increase of intracellular concentration of DDP (Lou et al., 2016). MVP, a known lung resistance protein, is a major component of a ribonucleoprotein organelle called vault, and which has been implicated in multiple drug resistance of cancer cells. Thus, we first investigated whether the application of YPFS_+GF_ in cultured A549/DDP cells could further increase the intracellular concentration of DDP. As expected, the intracellular concentration of DDP was significantly increased to about 25–50%, as compared to application of YPFS or Gingkgo Folium extract (**Figure 3A**). The treatment of MK571, an inhibitor of multidrug resistance-associated protein (MRP1), served as a positive control. DNA damage is one of the consequent events after an increase of intracellular concentration of DDP. To support this notion, the level of double strand DNA marker, γH2AX, was determined in the culture. Application of YPFS_+GF_ increased the level of γH2AX in DDP-treated A549/DDP cells (**Supplementary Figure 5**).

**FIGURE 5:**
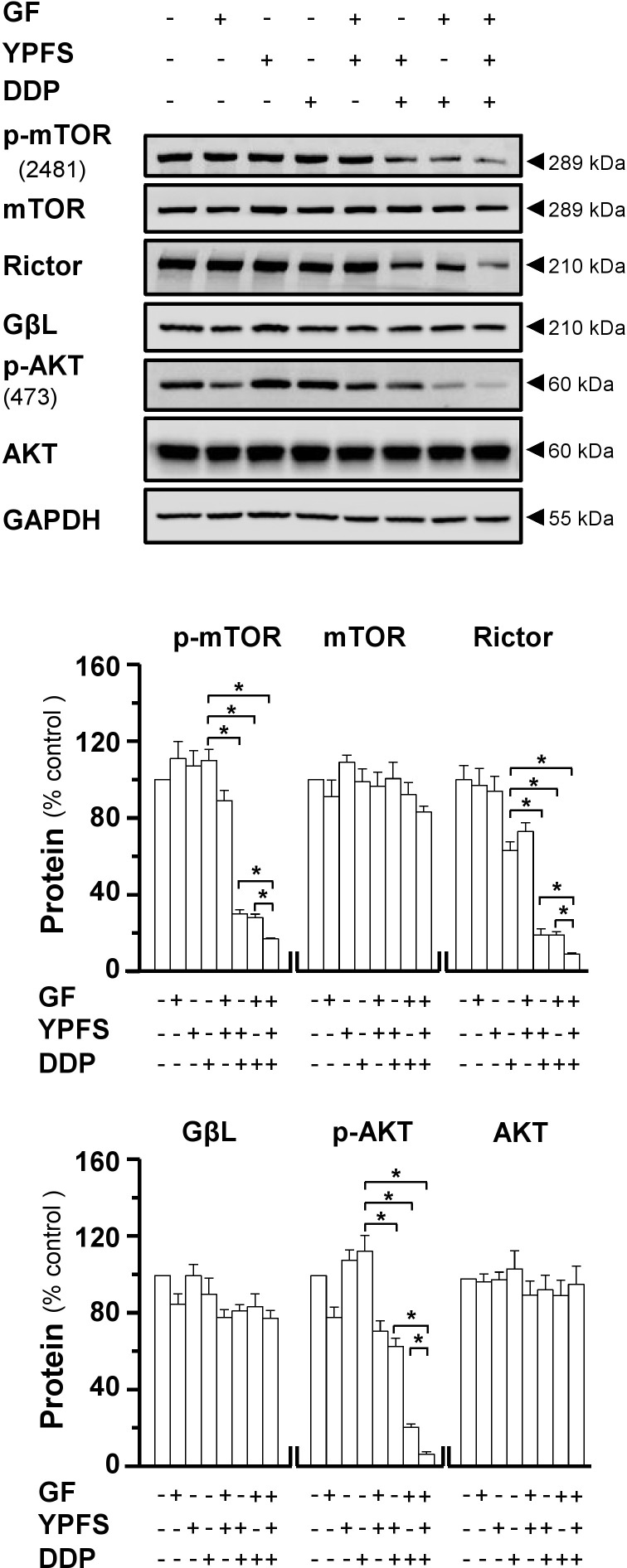
YPFS_+GF_ disrupts mTORC2 complex in A549/DDP cells. Cultured A549/DDP cells were treated with DDP, YPFS, GF, YPFS_+GF_, and DDP with or without YPFS, GF or YPFS_+GF_ for 48 h, as in **Figure 4**. Western blot was applied to analyze the amount of p-mTOR (∼289 kDa), mTOR (∼289 kDa), Rictor (∼210 kDa), GβL (∼37 kDa), p-AKT (∼60 kDa), and AKT (∼60 kDa) (upper panel). Expression of GAPDH (∼37 kDa) served as a control. Quantitation of protein expression is shown in lower panel. *n* = 3. ^∗^*p* < 0.05.

This anti-drug resistance phenomenon was concomitant by a decline on mRNA expression of MVP. YPFS, or Gingkgo Folium extract, significantly decreased the DDP-induced elevation on MVP mRNA, while YPFS_+GF_ could further decline this mRNA expression (**Figure 3B**). To determine the post-transcriptional regulation of MVP, western blot was conducted to observe the protein level of MVP. The DDP-induced up regulation of MVP protein (∼4 folds) was suppressed in the present of YPFS_+GF_: the suppression brought the level of MVP almost back to the background level (**Figure 3C**). These data suggested that YPFS_+GF_ could modulate MVP expression in both transcriptional and translational levels.

WT1 is a transcription factor that regulates the transcription of MVP. Here, the activation of WT1 transcriptional factor, after YPFS_+GF_ application, was determined. After application of YPFS_+GF_, a robust reduction of WT1 expression was found in DDP-treated A549/DDP cell: this reduction was much obvious than that of YPFS (**Figure 4A**). To further confirm the activity of WT1, the nuclear translocation of WT1 in the drug-treated A549/DDP cells was determined using immunofluorescence staining (**Figure 4B**). In cultured A549/DDP cell, DDP notably elevated the nuclear accumulation of WT1 by over 50%. The elevation on nuclear translocation was attenuated by application of either YPFS or Gingkgo Folium; however, the robust reduction, at least by over 4-fold, was being observed in YPFS_+GF_-treated cells (**Figure 4C**). By ChIP/PCR assay, an increased binding of WT1 transcription factor to the MVP promoter was observed after application of DDP. The protein-DNA binding was significantly reversed by application of YPFS_+GF_ (**Figure 4D**). These data suggested that YPFS_+GF_ might modulate WT1 activity, and consequently reduced the expression of MVP. The final outcome was an increase in intracellular concentration of DDP in cultured A549/DDP cells.

### YPFS_+GF_ Inhibits mTORC2/AKT Activity

Major vault protein induces cancer cell drug resistance not only due to intracellular distribution of DDP, but also contributes to regulation on mTORC2/AKT axis. To address the possible role of YPFS_+GF_ in mTORC2 signaling, we determined the stability of mTORC2 under application of DDP in absence or presence of herbal extracts. The expressions of major components of mTORC2, i.e., mTOR, Rictor and GβL, were determined by western blot. The expression of Rictor was notably decreased in treatment of YPFS_+GF_ in DDP-treated A549/DDP cells, as well as phosphorylation of mTOR at Ser2481 site. However, a slightly decrease was revealed under the application of either YPFS or Gingkgo Folium in DDP-treated cells. Rictor expression and phosphorylation level of mTOR on Ser2481 represent the intact function of mTORC2. The reduction on these two protein targets, induced by YPFS_+GF_, suggested that YPFS_+GF_ could disrupt the function of mTORC2 in DDP-treated A549/DDP cells (**Figure 5**). It is known that mTORC2 could phosphorylate AKT at Ser473 sites through phosphorylating mTOR at Ser2481 site (Sarbassov et al., 2005). Thus, we further determined the phosphorylation level of AKT at Ser473. Similarly, the declined level was observed in co-treatment of DDP + herbal extract. Again, YPFS_+GF_ showed the best induction (**Figure 5**).

**FIGURE 6:**
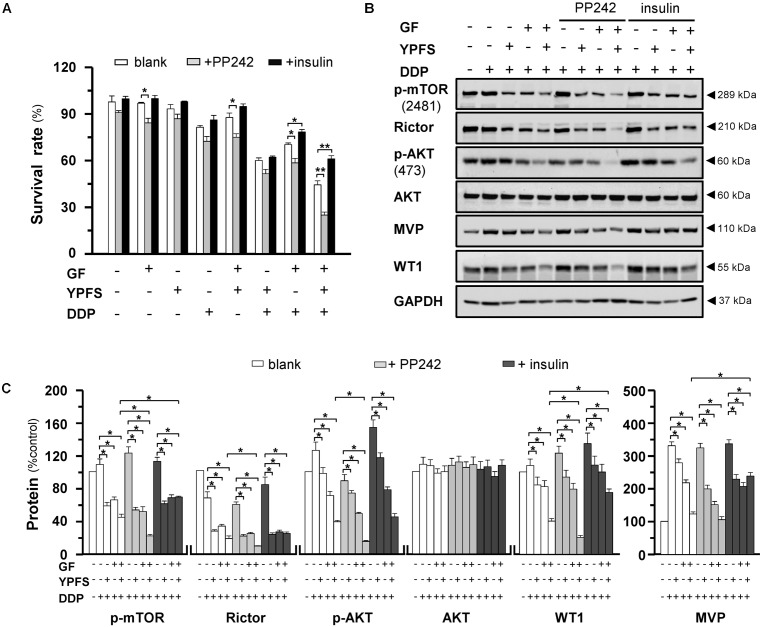
The YPFS_+GF_-mediated disruption on mTORC2 is promoted by PP242 and is reversed by insulin in A549/DDP cells. **(A)**: Cultured A549/DDP cells were treated with DDP, YPFS, GF, YPFS_+GF_, and DDP with YPFS, GF or YPFS_+GF_, the concentration as in **Figure 4**, in absence or presence of PP242 (125 nM) or insulin (2 μM) for 48 h. Cell viability was detected by MTT assay. Values are in percentage of cell viability. **(B)**: Cultured A549/DDP cells were treated with DDP (10 μM), DDP + YPFS, DDP + GF and DDP + YPFS_+GF_ with or without PP242 (125 nM) or insulin (2 μM) for 48 h. Protein amounts of p-mTOR (∼289 kDa), Rictor (∼210 kDa), p-AKT (∼60 kDa), AKT (∼60 kDa), MVP (∼110 kDa) and WT1 (∼55 kDa) were detected by western blot. Expression of GAPDH (∼37 kDa) served as a control. **(C)**: Quantitation of protein expression was calibrated from **(B)**. Results are expressed as the mean ± SEM, *n* = 3. ^∗^*p* < 0.05.

Next, we modulated the process of AKT at Ser473 phosphorylation by chemical approach. Torkinib (PP242) is a selective mTOR inhibitor, which specially inhibits the mTORC2-induced AKT at Ser473 phosphorylation; while insulin activates mTORC2 signaling. Application of PP242 in cultured DDP-treated A549/DDP cells could promote the YPFS_+GF_-mediated inhibition effect. In a reciprocal manner, the mTOR phosphorylation at Ser2481, triggered by insulin, was notably reversed the YPFS_+GF_-suppressed cell proliferation (**Figure 6A**). This phenomenon was concomitantly with the changes on Rictor expression, as well as the phosphorylation levels of mTOR Ser2481 and AKT Ser473. The reduction on these three targets, triggered by YPFS_+GF_, could be altered significantly by using either insulin or PP242. PP242 promoted the YPFS_+GF_-mediated inhibition effect, including mTOR phosphorylation at Ser2481, AKT at Ser473 phosphorylation and expression of Rictor to a further degree at least by 2-fold (**Figures 6B,C**). Application of insulin reversed YPFS_+GF_-mediated inhibition on p-mTOR; however, the effect was slightly revealed in p-AKT and Rictor (**Figures 6B,C**). Of particular interest, insulin largely reversed the YPFS_+GF_-induced suppression on WT1 and MVP, and PP242 slightly promoted the decrease on expressions of WT1 and MVP (**Figures 6B,C**). These data suggested that the new herbal formula YPFS_+GF_ could promote the disruption on mTORC2 signaling, consequently decreased the phosphorylation on AKT Ser473, and this process might be modulated by the suppression on WT1/MVP axis.

## Discussion

**FIGURE 7:**
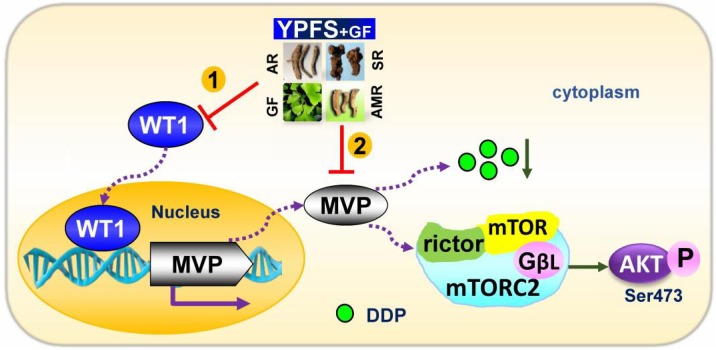
Model of YPFS_+GF_ promoting YPFS-induced sensitized effect on DDP-treated A549/DDP cells. YPFS_+GF_ suppresses the WT1/MVP axis in YPFS+DDP-treated A549/DDP cells, consequently increases the intracellular concentration of DDP, as well as inhibition on mTORC2/AKT axis. Finally, YPFS_+GF_ promotes the sensitized effect of YPFS in DDP-treated A549/DDP cells.

Here, we applied additional herbal extract in combining with YPFS aiming to improve the efficacy in anti-drug resistance in cultured A549/DDP cells. The re-formulated YPFS_+GF_, a combination of YPFS and Gingkgo Folium extract, showed a robust sensitized effect on DDP-induced resistance in cultured A549/DDP cells. This sensitized effect of YPFS_+GF_ was characterized by several parameters: (i) YPFS_+GF_ promoted cell cytotoxicity; (ii) YPFS_+GF_ increased apoptosis; (iii) YPFS_+GF_ elevated down regulation on WT1/MVP axis, consequently up regulated the intracellular concentration of DDP; (iv) YPFS_+GF_ declined activity of mTORC2/AKT in reducing the drug resistance (see **Figure 7**). This is the first time to reveal a novel formula from Chinese herbal medicine to have promising sensitized effect on DDP-treated A549/DDP cells. Besides, both YPFS and Ginkgo Folium are general considered as safe herbal medicine.

The decreased intracellular concentration of DDP due to efflux by transporters is one of the major mechanisms responsible for DDP-induced drug resistance in cancer treatment (Amable, 2016). Application of YPFS_+GF_ in DDP-treated cultured A549/DDP cells caused a downregulation of MVP, which consequently increased the intracellular DDP. MVP is a non-ABC transporter involving in intracellular DDP accumulation. The expression of MVP is specific to DDP-induced drug resistance in NSCLC, but not to other chemotherapeutic drugs, e.g., anthracyclins, etoposide or vinblastine. MVP is being considered as a drug resistant biomarker in NSCLC: because this is being up-regulated in many cancer cell lines, i.e., A549, Calu-3 and Calu-6. In line to this notion, the expression of MVP is positively related to poor prognosis in advanced NSCLC. The transcriptional regulation of MVP could be mediated by transcription factor WT1 via binding to the promoter element (Oji et al., 2004). In parallel, the expression of WT1 was up regulated under exposure of DDP in lung cancer, which therefore promoted the DDP-induced resistance in cancer patients (Wu et al., 2014). Here in cultured A549/DDP cells, application of DDP promoted WT1/MVP axis, which was characterized by an increase on WT1 protein expression, nuclear translocation, binding to MVP promoter, as well as induction of MVP expression. This phenomenon was consistent with previous study that DDP-induced resistance in A549 cell was correlated with high activation on WT1/MVP axis (Wu et al., 2014). Our previous study demonstrated that YPFS by itself promoted intracellular accumulation of DDP in cultured A549/DDP (Lou et al., 2016). Here, the application of YPFS_+GF_ further increased the intracellular DDP concentration, which indicated that the new formula, as compared with YFPS, could further inactivate WT1/MVP axis in DDP-treated A549/DDP cells.

Major vault protein plays multiple roles in DDP-induced resistance in NSCLC through several processes. Besides as a drug transport, MVP triggers cell death signaling pathways, e.g., mTOR/AKT pathway, to evade cell death (Park, 2012; Tang et al., 2013). Here, we hypothesized that the anti-cancer effect of YPFS_+GF_ could be partly accounted by regulation of MVP. MVP was shown to enhance cell survival by inducing stabilization of mTOR/AKT axis and hyper-activation of AKT: this process however could be reversed by mTOR inhibitors (Lötsch et al., 2013). AKT is a substrate of mTORC2, which phosphorylates AKT at hydrophobic motif site (Ser473). The down regulation of Rictor and the phosphorylation of mTOR Ser2481 could serve as biomarkers of mTORC2 complex disruption (Guertin et al., 2006; Copp et al., 2009). The decrease of phosphorylation of p-mTOR at Ser2481, p-AKT at Ser473 and Rictor expression were revealed in application of YPFS_+GF_ in DDP-treated A549/DDP cells; application of other herbal extracts however did not show such regulation. In addition, the PP242-blocked AKT phosphorylation at Ser473 (Hoang et al., 2012) could attenuate the YPFS_+GF_-induced down regulation of mTORC2/AKT: this result was in line with the aforementioned effect of YPFS_+GF_. Intriguingly, the activation of mTORC2 by insulin promoted the expressions of WT1 and MVP in DDP+YPFS_+GF_-treated A549/DDP cells. It has been reported that mTORC2 could be activated by insulin and phosphorylated AKT. The activated AKT could promote the expression of WT1 (Cybulski et al., 2009). In addition, a positive feedback loop between WT1 and AKT has been proposed in lung cancer cells (Wang et al., 2013). Thus, the activation of AKT, induced by insulin, resulted in a high level of WT1 expression that consequently increased MVP expression and finally reversed anti-resistant effect of YPFS_+GF_. These phenomena indicated that the inactivation of mTORC2/AKT, WT1/MVP could be crucial in YPFS_+GF_-mediated anti-resistant effect on DDP.

As shown here, the inclusion of Ginkgo Folium strongly enhanced the anti-cancer effect of YPFS. This specific addition is not fully resolved here. According to Chinese medicinal theory, a prescription of herbal formula is based on herb–herb interaction: this principle has not been changed today. Ginkgo Folium is a living fossil plant, which has been used in record by over few thousands of years (Cao et al., 2017). The extract of Ginkgo Folium, e.g., EGb761, is one of the most popular botanical supplements today. Different lines of evidence indicated that Ginkgo Folium exhibited anti-cancer effects. The Ginkgo Folium-treated sarcoma 108 (S180)-bearing mice significantly showed a reduced tumor weight (Feng et al., 2009). In addition, Ginkgo triggered DNA damage and cell cycle perturbation in HepG2 hepatocellular carcinoma cells (Zhang et al., 2015). The major constituents of Ginkgo Folium, e.g., flavonoid and terpenoid, were considered to inhibit the tumor growth (DeFeudis et al., 2003). Quercetin, kaempferol, isorhamnetin, ginkgolide and bilobalide are the most studied constituents within Ginkgo Folium for cancer therapy. Indeed, ginkgetin, a bioflavonoid in Ginkgo Folium, has been shown to have anti-cancer activity in prostate cancer (You et al., 2013), renal carcinoma (Ren et al., 2016) and osteosarcoma (Xiong et al., 2016). Moreover, the treatment of ginkgetin in lung cancer cells bearing mice could markedly reduce the tumor size, and which was proposed to be mediated by an autophagy-mediated cell death (Lou et al., 2017). Ginkgo biloba is widely cultivated in China, which is the largest producer of ginkgo biloba extract in the world. Thus, it is possible to use the extract of leaves for adjuvant therapy for cancer treatment, especially in Chinese medicine. The study of this novel formula will provide a clue on application of herbal extract, as well as to develop a non-canonical method in NSCLC treatment.

## Conclusion

In conclusion, the novel formula YPFS_+GF_ exhibited better sensitized effect on DDP-induced resistant in A549/DDP cells. This effect is targeting on the key mechanism of DDP resistant, including intracellular drug concentration, drug transporter MVP, and anti-apoptosis pathway mTORC2/AKT. The usages of YPFS and Ginkgo Folium have long historical record, which have been demonstrated to be safe. Herbal medicine is one of the main adjuvant therapies in NSCLC, and this result supports the prescription of traditional Chinese medicine in the cancer treatment.

## Author Contributions

JS-L, Z-YZ, and KT conceived and designed the experiments and wrote the main text. J-SL and YT-X performed the *in vitro* studies. H-YW, X-PK, and PY carried out the data collection and supervised the study. TD contributed reagents, materials, and analytic tools. All authors read and approved the final manuscript.

## Conflict of Interest Statement

The authors declare that the research was conducted in the absence of any commercial or financial relationships that could be construed as a potential conflict of interest.

## Abbreviations

DDP: cisplatin
ICP-OES: inductively coupled plasma optical emission spectrometry
mTORC2: mammalian target of rapamycin complex 2
MVP: major vault protein
NSCLC: non-small cell lung cancer
YPFS: Yu Ping Feng San
YPFS_+GF_: YPFS + Gingkgo Folium extract.
