# Supplementary material for: The WT1/MVP-Mediated Stabilization on mTOR/AKT Axis Enhances the Effects of Cisplatin in Non-small Cell Lung Cancer by a Reformulated Yu Ping Feng San Herbal Preparation
Source: Front Pharmacol. 2018 Aug 7;9:853. doi: 10.3389/fphar.2018.00853 (PMC6090061; doi:10.3389/fphar.2018.00853)
Supplement: Supplementary file 1 [file Table_1.DOCX]

**Supplementary data**

**
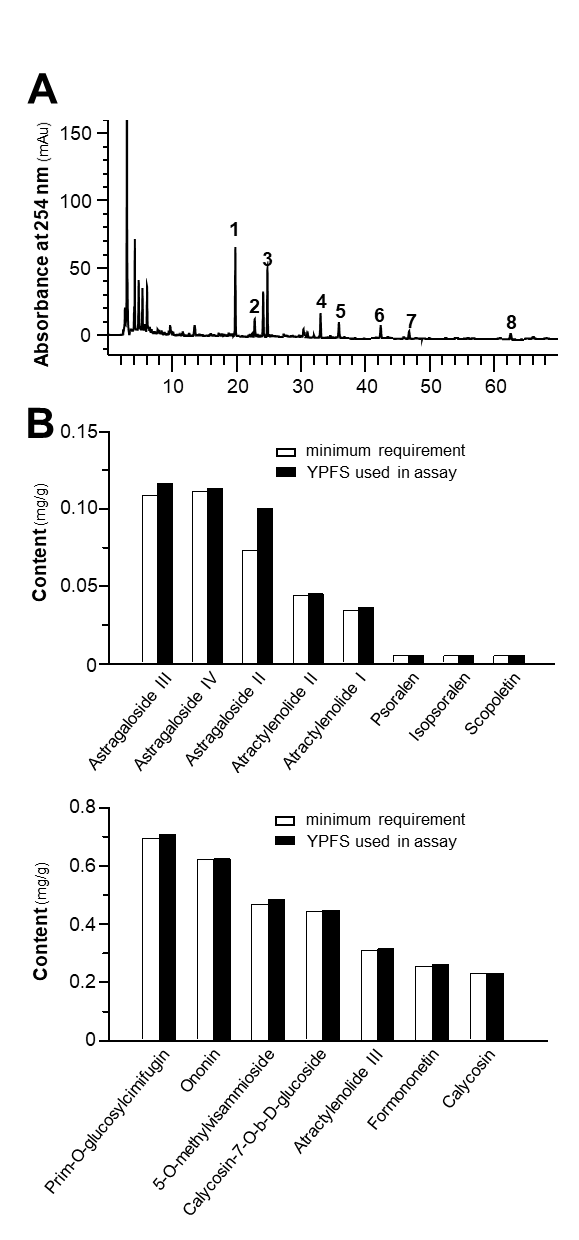
**

**Figure S1. The quality control of YPFS extract.**

**(A):** Typical HPLC fingerprint of YPFS at 254 nm. Forty mg/mL YPFS was subjected to HPLC analysis, and the chemical fingerprint was revealed by a DAD detector. prim-O-glucosylcimifugin (1), calycosin-7-O-β-D-glucoside (2), 5-O-methylvisammioside (3), ononin (4), calycosin (5), psoralen (6), formononetin (7), atractylenolide I (8) were identified in the HPLC fingerprint of 254 nm. Representative chromatograms are shown, *n* = 3. **(B):** The contents of 15 chemical markers in YPFS extract. YPFS was dissolved in methanol (1 mg/mL) and filtered. YPFS extract was subjected to the rapid resolution liquid chromatography/tandem mass spectrometry in negative or positive ion mode. Two suitable transition pairs were chosen for acquisition in multiple reaction monitoring. White column represents the minimum contents requirements of 15 markers in YPFS extract, the black column represents the amount of 15 markers in YPFS that used in current study. Values are expressed in mg/g from dried extract of YPFS, *n* = 4.


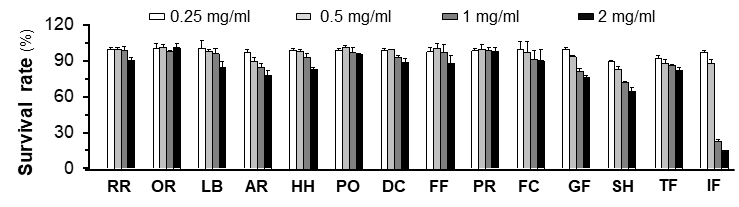


**Figure S2. The inhibition of 14 herbal extracts in proliferation of A549/DDP cells.**

Cells were seeded in 96-well plates (3 X 10^3^ cells/well) and allowed to adhere overnight. Cells were treated with various concentrations of herbal extracts (0.25, 0.5, 1 and 2 mg/mL). Values are in percentage of cell viability. Each point represents the mean ± SEM, *n* = 3. RR: Rehmanniae Radix, OR: Ophiopogonis Radix, LB: Lilii Bulbus, AR: Adenophorae Radix, HH: Houttuyniae Herba, PO: Polygonati Odorati Rhizoma, DC: Dendrobii Caulis, FF: Farfarae Flos, PR: Platycodonis Radix, FC: Fritillariae Cirrhosae Bulbus, GF: Ginkgo Folium, SH: Selaginellae Herba, TF: Taxus Folium, IF: Ilex Folium


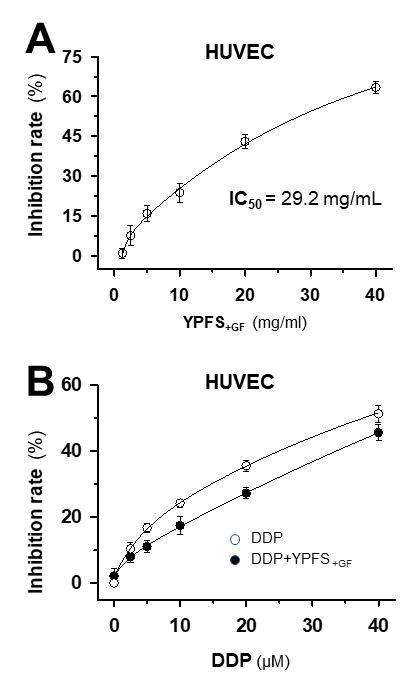


**Figure S3. YPFS_+GF_ slightly reduced DDP-induced cytotoxicity in HUVEC cells.**

**(A):** The IC_50_ of YPFS_+GF_ in HUVEC cells. Cells were seeded in 96-well plates (5 X 10^3^ cells/well) and allowed to adhere overnight. Cells were treated with various concentrations of YPFS_+GF_ (1.25, 2.5, 5, 10, 20 and 40 mg/mL) for 48 hours, the weight ratio of YPFS and GF in YPFS_+GF_ is 4:1.

**(B):** Cells were seeded in 96-well plates (5 X 10^3^ cells/well) and allowed to adhere overnight. Cells were treated with various concentrations of DDP (2.5, 5, 10, 20 and 40 μM) with or without YPFS_+GF_ (1 mg/mL + 0.25 mg/mL) for 48 hours. Each point represents the mean ± SEM, *n* = 3.


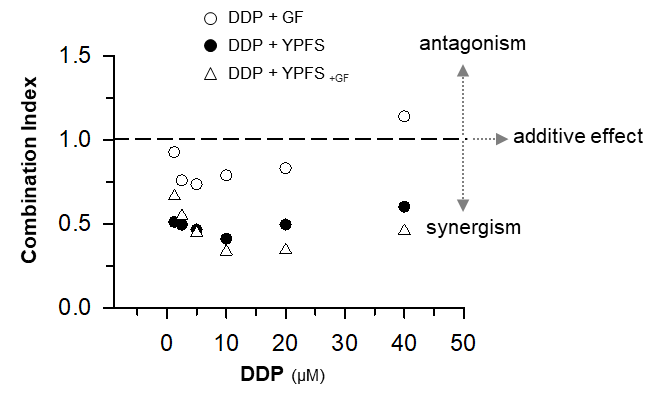


**Figure S4. Combination index of DDP+YPFS, DDP+GF, and DDP+YPFS_+GF_**

The Combination Index (CI) was calculated using CompuSyn software. In cultured A549/DDP cells, DDP were applied at different concentration, i.e. 1.25, 2,5, 5, 10, 20, 40 µM. White circle represents DDP combined with GF at 0.25 mg/mL. Black circle represents DDP combined with YPFS at 1 mg/mL. White triangle represents DDP combined with YPFS_+GF_ (1 mg/mL + 0.25 mg/mL). CI values <1, =1 and >1 indicate synergism, additive and antagonism effect, respectively.


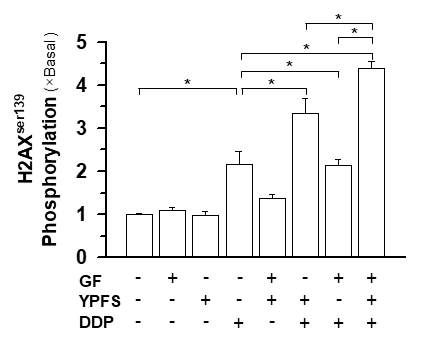


**Figure S5. YPFS_+GF_ promotes YPFS-mediated sensitized effect in DDP-induced DNA damage on A549/DDP cells.**

Quantification of DNA damage by measuring H2AXSer^139^ phosphorylation, determined by western blotting, in A549/DDP cells. Cultured A549/DDP cells were treated with YPFS (1 mg/mL), GF (0.25 mg/mL) or YPFS_+GF_ (1 mg/mL + 0.25 mg/mL) and DDP (10 μM) in absence or presence of YPFS (1 mg/mL), GF (0.25 mg/mL) or YPFS_+GF_ (1 mg/mL + 0.25 mg/mL) for 48 hours. Values are relative amount in fold of change (X Basal) to control (no drug treatment). Results are expressed as the Mean ± SEM, *n* = 3. **p* < 0.05.
